# Insurer Size and Negotiated Hospital Prices: Insights From the Affordable Care Act in Arkansas

**DOI:** 10.1002/hec.70022

**Published:** 2025-08-01

**Authors:** Jee‐Hun Choi

**Affiliations:** ^1^ Economics Department College of Business Lehigh University Bethlehem Pennsylvania USA

**Keywords:** affordable care act, buyer size effect, insurer–hospital negotiation, medicaid expansion, price negotiation

## Abstract

This paper examines the role of insurer size in price negotiations between commercial health insurers and hospitals in the United States. The empirical analysis focuses on a dominant insurer in the Arkansas individual health insurance market that experienced a size increase due to a policy change. Under the Affordable Care Act (ACA), Arkansas expanded its Medicaid program, but unlike other expansion states, it used individual plans—a private insurance option generally not designed for Medicaid—to provide coverage to newly insured beneficiaries. This unique policy nearly doubled the insurer's individual plan enrollment after the ACA was implemented. Admission‐level regression analysis reveals that the insurer's hospital inpatient prices for individual plans decreased by 16.7% following the expansion. Consistent with the predictions from bargaining models, the findings suggest that the insurer's increased bargaining leverage due to its larger size is the primary mechanism behind the price reduction.

## 1 Introduction

It is widely recognized across many industries that larger buyers enjoy a bargaining advantage in bilateral negotiations with suppliers.^1^ The theoretical rationale is that large buyers guarantee suppliers a substantial surplus from the negotiation, prompting them to accept lower prices.^2^ This principle is particularly relevant in U.S. healthcare markets, where bilateral negotiations between insurers and medical providers play a central role in determining the prices of healthcare services. As most commercial health plans operate as managed care plans with exclusionary provider networks, these negotiations determine two key outcomes: whether a provider will be included in the insurer's network and, if so, the insurer's reimbursement rates for the in‐network provider. Managed care plans steer beneficiaries to in‐network providers through various methods, such as different cost‐sharing arrangements and referral requirements for in‐network versus out‐of‐network providers. As a result, insurers are able to secure rate discounts from in‐network providers based on the surplus those providers derive from network participation.^3^ This dynamic makes insurer size a crucial factor in price negotiations, as larger insurers offer access to a greater volume of patients and, consequently, a larger potential surplus for providers. Prior empirical studies have consistently shown that an insurer's size or market share is a key predictor of negotiated prices across a range of healthcare services.^4^

This paper investigates whether the insights from existing empirical studies, most of which rely on cross‐sectional size variation across insurers, can be extended to instances of insurer size increases. The within‐insurer, cross‐time variation in size is also an important dimension to consider, given that major private insurers have steadily grown over time. According to Schoen and Collins (2017), the total enrollment of the Big Five commercial insurers (i.e., UnitedHealthcare, Anthem, Aetna, Cigna, and Humana) increased by 22.5% nationwide between 2010 and 2016. Additionally, the revenues of the six largest for‐profit commercial health insurers increased from 10% of the total U.S. health spending in 2011 to 30% in 2023 (Owens 2024). Nevertheless, empirical studies exploring how, and through what mechanisms, negotiated prices respond to increases in insurer size have been limited, presumably due to the endogeneity of size changes.

To fill this gap, I examine the effect of an insurer size increase resulting from the Affordable Care Act (ACA) in Arkansas. As of 2025, Arkansas is one of 41 states (including the District of Columbia) that have expanded eligibility for Medicaid—a means‐tested public health insurance program primarily for low‐income populations—under the ACA. Like most expansion states, Arkansas expanded its Medicaid program to childless adults and parents who were previously ineligible. However, unlike most expansion states—which provided coverage to newly insured Medicaid beneficiaries through Medicaid managed care (MMC) plans, designed exclusively for Medicaid recipients—Arkansas used a new type of individual plan introduced by the ACA. As with existing individual plans, these ACA individual plans are private insurance options typically intended for those not covered by employer‐sponsored plans or public programs like Medicaid. Due to this unique approach, approximately 250,000 new beneficiaries entered the Arkansas individual market in the first 2 years of the expansion (ACHI 2016). Moreover, the majority of these beneficiaries were enrolled in Arkansas Blue Cross Blue Shield (BCBS), the largest incumbent insurer in the market. As a result, BCBS individual plan enrollment surged by 186% between 2013 and 2015, according to Health Insurance & Managed Care Indicators by the Kaiser Family Foundation.

Given this context, I conduct a regression analysis using hospital claims data for inpatient services from the Arkansas All‐Payer Claims Database (APCD) between 2013 and 2015 to examine the impact of BCBS's ACA‐induced enrollment growth in individual plans on negotiated hospital prices. The analysis reveals that, on average, BCBS negotiated hospital prices that were 16.7% lower for its ACA individual plans compared to its pre‐ACA (i.e., existing) individual plans.

In subsequent analyses, I provide evidence suggesting that the ACA‐induced enrollment growth is the primary driver of BCBS's price reductions for individual plans. First, I find that these price reductions are greater in counties where a larger share of the population became eligible for Medicaid through the ACA—which is the main source of BCBS's enrollment growth. Specifically, a one‐percentage‐point increase in the share of the eligible population is associated with a 2.3‐percentage‐point larger price reduction. Additionally, an analysis of price changes across different types of services reveals that the reduction is more pronounced for services frequently utilized by newly insured Medicaid beneficiaries. These empirical findings align with the theoretical prediction that BCBS's price reductions are larger in areas and for services where its size increase generates greater potential surpluses for hospitals.

Furthermore, BCBS's price reductions for Arkansas hospitals do not extend to two in‐network hospitals in a neighboring state that have a reasonable number of BCBS individual plan claims. Unlike Arkansas hospitals, BCBS's size increase is not expected to confer a bargaining advantage in its negotiations with these out‐of‐state hospitals—even though they are in‐network—because their primary patient base consists of residents of their own states. The findings from out‐of‐state hospitals are consistent with this theoretical prediction.

In contrast, I find no substantial evidence supporting alternative explanations for the observed price difference. First, BCBS's ACA and existing individual plans share key features relevant to insurer–hospital price negotiations, such as managed care type and hospital network composition. Thus, differences in plan design do not fully explain the price gap. Second, the ACA introduced provisions—such as guaranteed issue, which prohibited insurers from denying coverage based on pre‐existing conditions—that may have increased the risk level of individual plan pools, thereby incentivizing insurers to reduce costs in their individual plans. However, these regulations do not inherently strengthen an insurer's bargaining position, as they do not increase hospitals' surplus from negotiation—an essential factor for accepting lower prices. If these regulations were the primary mechanism, smaller price reductions would be expected among hospitals with significant market power, since those hospitals are better positioned to resist insurers' efforts to negotiate lower rates. Yet, my analysis of hospitals with potentially greater market power—namely, large and system‐affiliated hospitals—shows that their price reductions are not smaller. Finally, it is also unlikely that factors within the hospital sector (e.g., market concentration) explain the price difference because no significant changes occurred during this period, as shown in Supporting Information S1: Table A6.

The findings of this study align with previous studies examining how insurer size impacts price negotiations between insurers and providers in U.S. healthcare.^5^ While most studies have utilized cross‐sectional variations in insurer size to investigate its role in price negotiation, my findings contribute to this literature by empirically demonstrating that the primary mechanism behind the size effect—how much provider surplus is secured due to a size increase—also holds in a time‐series setting, particularly for hospital prices.

My findings also contribute to the broader literature on the role of firm size in price negotiations. Many studies have examined the importance of relative firm sizes (i.e., market share) in bilateral price negotiations.^6^ My findings extend this literature by highlighting the importance of a firm's *absolute* size in price negotiations, particularly in expanding markets. Unlike in non‐expanding markets, where changes in absolute size often coincide with shifts in market share, an expanding market allows a firm's absolute size to grow independently of its relative size. My empirical setting in Arkansas illustrates this distinction: while BCBS's absolute size increased in the Arkansas individual market after the ACA rollout, its market share decreased as a new insurer entered the market. My research shows that growth in absolute size can confer a bargaining advantage, even without changes in market share or market concentration.

This insight is crucial for understanding the price effects of market expansion, particularly in sectors where consumer prices are determined through bilateral negotiations, such as pharmaceuticals and video streaming. This dynamic is particularly relevant for the U.S. public health sector, where privatized public health plan markets have consistently grown. For instance, the share of Medicare Advantage plan enrollees—Medicare plans offered by commercial insurers—increased from 19% in 2007 to 51% in 2023 among Medicare beneficiaries (Ochieng et al. 2023). My findings suggest that when expanding public health programs through private plans, policymakers should consider how the expansion affects market outcomes, such as healthcare prices, even in the absence of significant shifts in market concentration.

Finally, my work contributes to research on the impacts of Arkansas's unique approach of using individual plans for Medicaid expansion. Most prior studies have focused on its effects on demand‐side outcomes, such as health coverage rates, access to care, healthcare utilization, and health outcomes among Medicaid beneficiaries (Sommers et al. 2016, 2017; Goudie et al. 2020; Bollinger et al. 2021; Steenland et al. 2021; Self et al. 2021). These studies generally report that Medicaid expansion in Arkansas is associated with improvements in these outcomes. However, the evidence is mixed on whether using individual plans leads to different changes in these outcomes compared to providing Medicaid coverage through traditional Medicaid or MMC plans.^7^ My paper extends this literature by demonstrating that Arkansas's unique Medicaid expansion approach influenced not only demand‐side outcomes but also supply‐side dynamics, particularly price negotiations between hospitals and commercial insurers enrolling Medicaid beneficiaries. This also suggests that when designing or expanding public health programs using private plans, policymakers must consider the potential impacts on commercial insurers and supply‐side outcomes.

## 2 Institutional Background

### 2.1 Insurer–Hospital Price Negotiation

In the United States, commercial health insurance carriers negotiate reimbursement rates with hospital systems. Because most private insurance plans operate as managed care plans with exclusionary provider networks, hospital systems decide whether to join an insurer's network and, if they choose to do so, contract to establish predetermined rates. These in‐network rates are typically lower than out‐of‐network rates, as insurers negotiate discounts in exchange for steering patients to in‐network providers through cost‐sharing and referral mechanisms. These negotiations generally occur at the network level, even when insurers offer multiple plans (CBO 2022).

Section A1 of the Supporting Information S1 presents a bargaining model between insurers and hospitals. The model shows that the discount an insurer secures from in‐network hospitals depends on the surplus that each party derives from the agreement. The health economics literature commonly defines surplus as the profit gain each party realizes from including the hospital in the insurer's network (e.g., Ho 2009; Lewis and Pflum 2015; Gowrisankaran et al. 2015). For insurers, including a hospital in the network can increase consumers' willingness to pay for the plan, which in turn can boost enrollment and improve profitability. For hospitals, given their high fixed costs—such as equipment and infrastructure—higher patient volume resulting from network inclusion can also improve profitability (R. R. Roberts et al. 1999; Murray 2020).

Because a party's bargaining leverage increases when it can secure a larger surplus for its counterpart, its size plays a critical role in negotiations. Insurers with larger market shares or patient volumes have an advantage because they can direct more patients to providers. Conversely, dominant hospital systems also hold leverage, as insurers rely on them to build competitive networks. Empirical studies confirm that size and market share are key drivers of healthcare price variation (e.g., E. T. Roberts et al. 2017; Barrette et al. 2022).

However, even smaller insurers or providers can gain leverage if their characteristics generate substantial surplus for their counterparts. Insurers offering popular plans or demonstrating strong patient‐steering capabilities may secure favorable terms despite having a smaller market share. Similarly, providers offering specialized services or enjoying strong patient loyalty may hold bargaining power, as insurers depend on them to meet enrollee needs. Empirical studies, such as Sorensen (2003) and Wu (2009), support these predictions.

### 2.2 The Affordable Care Act

The ACA is a federal statute that introduced major changes to the U.S. healthcare system, primarily to expand insurance coverage and improve the functioning of individual insurance markets. It aimed to achieve these goals by (1) reforming individual plans and (2) expanding Medicaid eligibility. Signed into law in 2010, most provisions took effect in 2014.

Individual plans are private insurance options designed primarily for those without employer‐sponsored coverage or who are ineligible for public programs like Medicaid. The ACA reformed this market by introducing new individual plans (hereafter ACA individual plans), leading many insurers to discontinue or modify existing plans to comply with new regulations. While targeting the same population segments, ACA individual plans differ in three key ways from their pre‐ACA counterparts.

First, while existing individual plans were typically purchased directly from insurers, ACA individual plans are often bought via online platforms known as health exchanges. ACA plans offered on exchanges are called exchange plans or qualified health plans (QHPs), while those sold directly by insurers are called off‐exchange plans. Second, ACA individual plans—both exchange and off‐exchange—are subject to new regulations to improve their affordability and accessibility. For instance, insurers cannot deny coverage or charge higher premiums based on pre‐existing conditions or gender. While these regulations also apply to existing individual plans, some grandfathered plans remained exempt. Third, ACA individual plan enrollees qualify for income‐based premium and cost‐sharing subsidies. Specifically, exchange plan enrollees with household incomes between 100% and 400% of the federal poverty level (FPL) receive premium tax credits, whereas those on Silver‐tier exchange plans earning between 100% and 250% of the FPL also qualify for cost‐sharing reduction (CSR) subsidies.^8^ Table 1 summarizes the differences between the two types of individual plans.

**TABLE 1:** Comparing ACA and existing individual plans.

	Existing individual plans		ACA individual plans	
	Pre‐ACA years	Post‐ACA years	(Introduced in 2014)	
	(Before 2014)	(2014 and after)	Exchange	Off‐exchange
ACA regulations applied?	No	**Yes** ^a^	**Yes**	**Yes**
Premium and CSR subsidies available?	No	No	**Yes**	No
Used in Arkansas's ACA Medicaid expansion?	No	No	**Yes**	No
Share within AR ACA individual^b^ (2015)	—	—	95.1%	4.9%

^a^ Although ACA provisions generally applied to existing individual plans in post‐ACA years, certain grandfathered plans were exempt from these provisions.

^b^ The shares are calculated by the author using the enrollment data from the Arkansas APCD.

During the study period (2014–2015), three carriers offered ACA individual plans in Arkansas, with 95% of enrollees covered by exchange plans (Table 1). Among them, Arkansas BCBS was the largest carrier and had offered individual plans before the ACA. Like other carriers, BCBS's pre‐ACA plans stopped enrolling new beneficiaries once ACA individual plans became available.

The ACA also expanded Medicaid, a means‐tested public health program that covers low‐income individuals. As of 2025, Arkansas is one of 41 states (including the District of Columbia) to have adopted this expansion, extending coverage to parents with household incomes between 17% and 138% of the FPL and to childless adults with household incomes up to 13% of the FPL (referred to as ACA Medicaid beneficiaries). However, unlike other states, Arkansas obtained a Section 1115 waiver from the Centers for Medicare & Medicaid Services (CMS) to provide coverage to newly eligible Medicaid enrollees through ACA individual plans rather than through traditional Medicaid or MMC plans.^9^ To ensure parity, CMS required Arkansas to subsidize premiums and out‐of‐pocket costs for ACA Medicaid enrollees. As a result, premiums and deductibles were waived for all ACA Medicaid beneficiaries, and cost‐sharing was subsidized, with amounts varying by income. For inpatient admissions, beneficiaries with household incomes below 100% of the FPL had no cost‐sharing, while those with incomes between 100% and 138% of the FPL were subject to a $140 copayment per admission, regardless of their plan choice (Bachrach et al. 2015).

## 3 Empirical Setting and Data

### 3.1 Study Context and Scope

The empirical analysis in this study examines how BCBS's hospital prices changed following the implementation of the ACA in 2014. Two details regarding the scope of my analysis are worth noting.

First, the study period spans from 2013 to 2015 for two primary reasons. The Arkansas APCD healthcare claims and enrollment data—the main dataset—are only available starting in 2013. Additionally, the increase in BCBS enrollment due to the ACA was most pronounced in the years immediately following its implementation, making these initial post‐ACA years the most relevant for analyzing the direct effect of enrollment growth on price negotiations.

Second, my analysis focuses on inpatient services, using the total negotiated amount (i.e., allowed amount) at the admission level as the primary measure for comparing hospital prices. The primary motivation for this approach is the availability of diagnosis‐related group (DRG) codes assigned to admissions. DRG codes classify hospital admissions based on diagnosis and severity to determine the resources required. Each of the approximately 760 DRG codes is associated with a DRG weight that reflects the relative resource required for an admission with that code, with higher values indicating greater resource needs. Thus, DRG codes and weights can be used to account for variation in negotiated amounts arising from differences in diagnoses and severity across hospital stays. Furthermore, prior research reports that DRG codes often serve as a basis for contract negotiations for inpatient admissions (Cooper, Craig, Gaynor, and Van Reenen 2019). Therefore, using the total negotiated amount while controlling for DRG codes at the admission level enables a comprehensive comparison of overall price levels across hospitals, which is the primary objective of this study.^10^

### 3.2 Data

The empirical analysis in this study primarily utilizes hospital inpatient claims and health insurance enrollment data from the Arkansas APCD for the study period, 2013 to 2015.

#### 3.2.1 Inpatient Claims Data

The inpatient claims data from the Arkansas APCD include details on hospital admissions, such as assigned DRG codes, length of stay, and National Provider Identifier numbers. The data also contain reimbursement information, including the negotiated amounts between insurers and hospitals (allowed amounts), as well as demographic details about patients, such as gender and age. The claims dataset is merged with the American Hospital Association (AHA) Survey data to obtain hospital characteristics.

I obtain the final sample of inpatient claims for this study by making the following restrictions. First, I focus on the inpatient claims for individual plans (both existing and ACA individual plans) made by general acute care hospitals. Second, I restrict the sample to claims from patients aged between 19 and 64 when they received the service because public insurance eligibility and healthcare utilization may differ significantly between the targeted population and children or the elderly. The claims sample also excludes claims without valid DRG codes and claims with outlier values for DRG weights or negotiated amounts.^11^ Finally, the sample excludes claims from non‐Arkansas hospitals.^12^ Supporting Information S1: Table A5 summarizes the steps for obtaining the final claims sample and how many observations were removed in each step. The final inpatient claims dataset includes 34,828 claims from 74 hospitals. Panel A of Table 2 presents the summary statistics for the key variables in the inpatient claims data. Panel B of Table 2 shows the summary statistics for hospital characteristics obtained from the AHA Annual Survey data.

**TABLE 2:** Summary statistics (2013–2015).

Variable	Mean	Std. Dev.
A. BCBS individual plan inpatient claims (34,828 claims)		
Distribution of patient age (shares)		
19–26^a^	0.154	0.361
27–34	0.181	0.385
35–44	0.168	0.374
45–54	0.230	0.421
55–64	0.266	0.442
Share of female patients	0.647	0.478
Share enrolled in ACA individual plans	0.841	0.366
Share covered by ACA Medicaid	0.653	0.476
Share of admissions in system‐affiliated hospitals	0.577	0.494
DRG weight	1.322	0.940
Length of stay (days)	4.326	3.409
Allowed amount ($1000)	9.439	8.008
Admission year (shares)		
2013	0.059	0.236
2014	0.398	0.490
2015	0.542	0.498
B. Hospitals^b^ (74 hospitals)		
Number of beds	134.1	143.7
Distribution of hospital ownership (shares)		
Government	0.149	0.358
Non‐profit	0.622	0.488
For‐profit	0.230	0.424
Share of teaching hospitals	0.027	0.163
Share of system‐affiliated hospitals	0.500	0.503
C. Individual market enrollment^c^ (646,630 member‐years)		
Distribution of enrollee age (shares)		
19–26^a^	0.190	0.392
27–34	0.198	0.399
35–44	0.208	0.406
45–54	0.216	0.411
55–64	0.188	0.391
Share of female enrollees	0.551	0.497
Share enrolled in ACA individual plans	0.661	0.473
Share covered by ACA Medicaid	0.524	0.499
Share enrolled in PPO plans	0.892	0.310
Share enrolled in Arkansas BCBS plans	0.746	0.436
Enrollment duration (member‐year)	0.762	0.303
Year (shares)		
2013	0.129	0.335
2014	0.371	0.483
2015	0.500	0.500

*Source:* (a) Overall: inpatient claims and enrollment data from the Arkansas APCD, (b) Hospital variables: American Hospital Association (AHA) Annual Hospital Survey, and (c) DRG weight: the list of MS‐DRGs from the Final Rule and Correction Notice for fiscal years 2013–2015 published by the CMS.

^a^ The upper limit of the youngest age group is set at 26, as the ACA extended the age threshold for dependent coverage to 26.

^b^ For each hospital, characteristics are taken from the most current year during the study period in which BCBS individual claims were available in the data.

^c^ (a) Enrollment duration is measured in member‐years: it takes a value of 1 if a beneficiary is enrolled for the entire year. (b) Statistics for all enrollment variables are weighted using enrollment duration, except for the variable “Enrollment duration.” (c) Since the enrollment dataset is at the year‐enrollee level, the variable “Year” indicates the year corresponding to a given observation.

#### 3.2.2 Enrollment Data

The enrollment dataset from the Arkansas APCD consists of plan beneficiaries enrolled in commercial insurance plans in Arkansas. It includes information on the market category of each plan (e.g., whether it is a group or individual plan), the type of managed care (e.g., Preferred Provider Organization (PPO) or Point of Service (POS) plan), enrollment duration, and demographic characteristics of beneficiaries, such as age and gender. The dataset also contains Health Insurance Oversight System (HIOS) ID numbers for ACA individual plans. These plan‐level identifiers are used to determine whether an enrollee is in an ACA individual plan and, if so, whether the enrollee is covered by Medicaid. To ensure consistency with the final inpatient claims sample, I restrict the enrollment data to non‐elderly adults living in Arkansas between 2013 and 2015 who were enrolled in one of the individual plans offered in the state's individual market. The final pooled enrollment dataset includes 646,630 member‐year enrollments. Panel C of Table 2 presents summary statistics for key variables in the enrollment data.

## 4 Changes in Enrollment and Hospital Prices

In this section, I examine the changes in individual plan enrollments and hospital prices for BCBS following the implementation of the ACA.

### 4.1 Enrollment Changes

**FIGURE 1:**
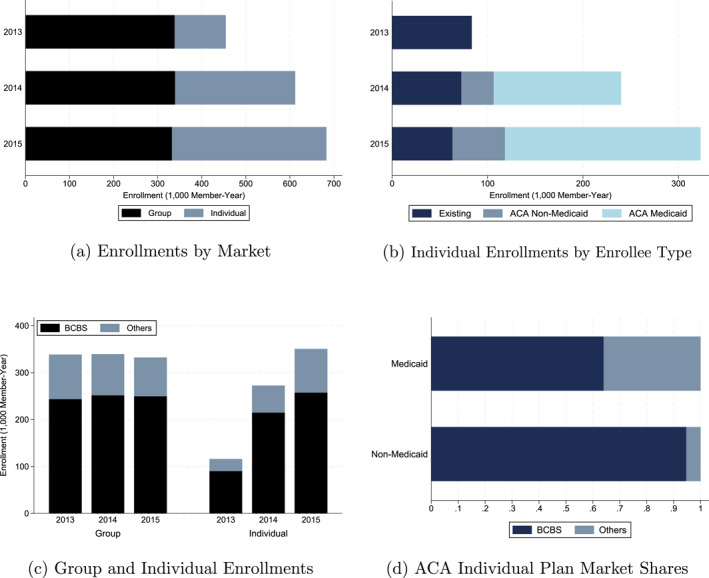
Health insurance enrollments in arkansas. (a) and (c): Health insurance & managed care indicators by KFF (https://www.kff.org/state‐category/health‐insurance‐managed‐care/insurance‐market‐competitiveness) (b) and (d): author's calculation using the enrollment data from arkansas APCD. Panel (a) presents the annual group and individual plan enrollments in Arkansas. Group plans include large and small group plans, and individual plans include ACA and pre‐ACA (i.e., existing) individual plans. Panel (b) breaks down the annual individual plan enrollments by enrollee type: existing individual plan enrollees, ACA plan enrollees covered by medicaid, and ACA plan enrollees not covered by medicaid. Panel (c) presents the annual group and individual plan enrollments for BCBS and other insurers combined. Panel (d) shows the market share of BCBS and the combined market share of other insurers broken down by Medicaid eligibility. KFF data are used for panels (a) and (b) because although Arkansas APCD data cover all individual plans, they do not cover all group plans. On the other hand, arkansas APCD data are used for panels (b) and (d) because the KFF data do not report individual plan enrollments by enrollee type and medicaid eligibility. even though the data sources are different, it can be seen that the overall trends of individual plan enrollment are consistent.

The implementation of the ACA in Arkansas led to a significant increase in health insurance enrollment, reducing the state's uninsured rate from 27.5% in 2013 to 15.6% in 2015 (ACHI 2016). This increase was primarily concentrated in the individual market, as the two key ACA measures—individual market reforms and Medicaid expansion using individual plans—were directly tied to individual coverage. Panel (a) of Figure 1 shows annual commercial insurance enrollments by market category, excluding self‐insured plans. Between 2013 and 2015, individual plan enrollments surged by 202%, while group plan enrollments experienced minimal change. Among the two ACA policy measures, Medicaid expansion through individual plans was the primary driver of this growth. Panel (b) of Figure 1 breaks down individual enrollments into existing individual plan enrollees (“Existing”), non‐Medicaid ACA individual plan enrollees (“ACA Non‐Medicaid”), and Medicaid ACA individual plan enrollees (“ACA Medicaid”). The figure shows that the growth of individual plan enrollment was primarily driven by an influx of ACA Medicaid beneficiaries, who accounted for 63% of total ACA individual plan enrollment in 2015.

Arkansas BCBS, the state's largest pre‐ACA insurer for individual and group plans, absorbed much of this growth, covering 73% of individual plan enrollees in 2015, as presented in Panel (c) of Figure 1. Consequently, BCBS's individual plan enrollment rose by 186% from 2013 to 2015, increasing from 5% to 14.3% of the non‐elderly adult population.^13^ Despite this growth, BCBS's market share in the individual plan segment declined from 78% to 73%, following the entry of a new insurer after the ACA was implemented.

Although ACA individual plans were offered by multiple carriers, three aspects of the ACA implementation in Arkansas made BCBS's enrollment growth largely exogenous to price negotiations. First, BCBS was the sole carrier of ACA individual plans in two service areas during the program's first year. Second, 65% of ACA Medicaid enrollees—the primary source of BCBS's enrollment growth—did not self‐select their plans in the first year.^14^ Instead, Arkansas auto‐assigned these individuals to plans, prioritizing smaller insurers to foster competition among carriers (CMS 2014). This process likely contributed to a higher market share for non‐BCBS plans among Medicaid enrollees than among non‐Medicaid enrollees, who were required to self‐select their plans (Figure 1d).

Third, while premium rates are generally closely tied to the reimbursement rates insurers pay providers (Trish and Herring 2015; Ho and Lee 2017), making insurance choices potentially endogenous to negotiated prices, this concern is mitigated in my setting due to the lack of premium and cost‐sharing variation across plans for Medicaid beneficiaries. As explained in Section 2.2, ACA Medicaid beneficiaries in Arkansas did not pay premiums because the state subsidized these payments. Moreover, those with household incomes below 100% of FPL—who accounted for 80% of Medicaid beneficiaries—faced no out‐of‐pocket costs at all. While beneficiaries with incomes between 100% and 138% of FPL did face cost‐sharing payments, the cost‐sharing schedules were uniform across all plans. Thus, BCBS's enrollment growth is unlikely to have been driven by Medicaid beneficiaries' preferences for the financial features of its plans.^15^

### 4.2 Changes in Hospital Prices

#### 4.2.1 Regression Model

In this section, I examine how hospital prices for BCBS individual plans changed after the ACA rollout. Since ACA individual plans were introduced by the ACA while existing individual plans remained active in the post‐ACA years—despite no longer accepting new enrollees—there are two types of individual plan claims in the post‐ACA period. Accordingly, I classify BCBS individual plan claims into the following three categories using indicators for ACA individual plans $\left({ACA\text{\_}Ind}_{i}\right)$ and for existing individual plans in the post‐ACA period $\left({Existing\text{\_}Ind\text{\_}Post}_{i}\right)$:

1. ACA individual plan claims, which exist only in the post‐ACA period (i.e., ${ACA\text{\_}Ind}_{i}\;=1$ and ${Existing\text{\_}Ind\text{\_}Post}_{i}\;=0$),
2. Existing individual plan claims in the post‐ACA years (i.e., ${ACA\text{\_}Ind}_{i}\;=0$ and $Existing\;{\text{\_}Ind\text{\_}Post}_{i}\;=1$), and
3. Existing individual plan claims in the pre‐ACA years (i.e., ${ACA\text{\_}Ind}_{i}\;=0$ and $Existing\;{\text{\_}Ind\text{\_}Post}_{i}\;=0$).

I then compare the prices of the two post‐ACA individual plans to pre‐ACA individual plan prices by estimating the admission‐level regression specification below, following approaches of prior studies (Wu 2009; Cooper, Craig, Gaynor, and Van Reenen 2019):

(1) $$\begin{array}{l} \log\left(P_{\mathrm{ijdt}}\right)=\;\beta_{0}+\beta_{1}\;{ACA\text{\_}Ind}_{i}+\beta_{2}\;{Existing\text{\_}Ind\text{\_}Post}_{i}+\beta_{l}\;\log\left({LOS}_{i}\right)\\ +\delta_{d}+\alpha_{j}+\beta_{t}\;{Year2014}_{i}+\varepsilon_{\mathrm{ijdt}}. \end{array}$$

P 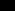 represents the total negotiated amount for admission i at hospital j with DRG code d in year t. $\delta_{d}$ captures DRG code fixed effects, which account for variation in negotiated amounts across diagnoses and severity, as discussed in Section 3.1. The model further includes $\log\left({LOS}_{i}\right)$, the logged length of stay for admission i (in days), as longer stays tend to incur higher costs. Additionally, $\alpha_{j}$ captures hospital fixed effects, and the coefficient for Year2014 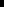 $\left(\beta_{t}\right)$ accounts for the fixed effect of claims from 2014 (as there are only two post‐ACA years). Finally, $\varepsilon_{\mathrm{ijdt}}$ represents mean‐zero shocks to prices not explained by the included covariates. I estimate Equation (1) using the claims sample described in Section 3.2 with standard errors clustered by hospitals.

**FIGURE 2:**
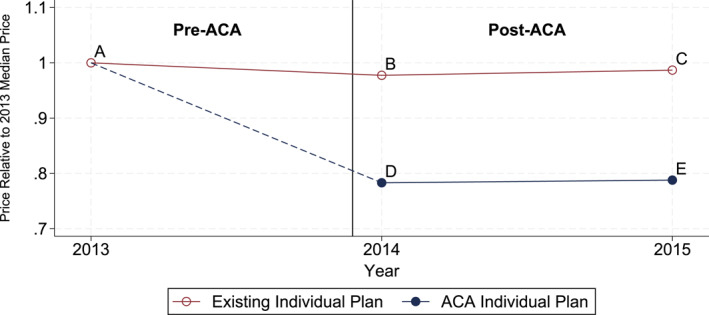
Example: Annual median prices of BCBS individual plans for DRG code 775. *Source:* Author's calculation using the inpatient claims data from the arkansas APCD.

As an illustrative example, Figure 2 plots the annual median negotiated price for hospital claims with DRG code 775, the most common code, separately for BCBS ACA and existing individual plans. Prices are expressed relative to the 2013 existing individual plan median price (“A”). Under this framework, $\beta_{1}$ and $\beta_{2}$ from Equation (1) measure how ACA individual plan prices (“D” and “E”) and existing plan prices (“B” and “C”) during post‐ACA years compare to pre‐ACA existing plan prices (“A”), after accounting for variations due to control variables.

#### 4.2.2 Estimation Results

Table 3 reports the estimation results for Equation (1). Column (1) shows the results using the entire set of claims and presents a stark contrast in the trend of negotiated prices between the two individual plans. The negotiated prices for BCBS's ACA individual plans are, on average, 18.2 log points (or 16.7%) lower than the pre‐ACA prices for existing individual plans. In contrast, the prices for existing individual plans barely changed after the ACA was implemented. One caveat is that the number of ACA plan claims is much larger than the number of existing plan claims; as such, the result may be due to different sets of hospitals used depending on plan type. Thus, I also estimate the model with a focus on the same combinations of DRG codes and hospitals as those present in pre‐ACA existing plan claims. Column (2) reports the estimation results and shows that they are similar to those in Column (1): BCBS ACA individual plan prices remain 18.9 log points (17.2%) lower than the pre‐ACA prices, whereas existing plan prices barely changed.

**TABLE 3:** Estimation results: Changes in negotiated prices.

	Dependent variable: log(negotiated price)	
	Entire claim set	Balanced hospitals and DRG codes
	(1)	(2)
ACA_Ind	−0.182***	−0.189***
	(0.025)	(0.027)
Existing_Ind_Post	0.0004	0.0002
	(0.008)	(0.008)
log(LOS)	0.158***	0.102***
	(0.018)	(0.020)
Year2014	−0.011***	−0.005
	(0.003)	(0.003)
Constant	8.734***	8.799***
	(0.038)	(0.042)
DRG FE	X	X
Hospital FE	X	X
Observations	34,828	15,541
*R*‐squared	0.909	0.937
Number of hospitals	74	40

*Note:* Robust standard errors, clustered by hospitals, are presented in parentheses (***p <0.01 **p <0.05 *p <0.1). All specifications estimate Equation (1). The key variables of interests are ACA_Ind and Existing_Ind_Post, whose coefficients compare the negotiated prices of BCBS ACA plans and existing plans in the post‐ACA period, respectively, in log points to existing plan prices during the pre‐ACA years, which serve as the reference group. Column (1) uses the full set of BCBS individual plan claims, while Column (2) focuses on the same combinations of DRG codes and hospitals as those observed in existing plan claims during the pre‐ACA period. For rows “DRG FE” and “Hospital FE,” an *X* indicates that the corresponding fixed effects are included in the specification.

Supporting Information S1: Table A2 in the Online Appendix shows that the estimation results presented in Table 3 remain robust when specifications use logged DRG weights (log(DRGW)), which quantify the resources required for each DRG code numerically, as a control variable instead of including DRG fixed effects. Additionally, Supporting Information S1: Table A2 demonstrates that the estimation results in Table 3 are robust when using a generalized linear model (GLM) in the estimation.^16^

#### 4.2.3 Discussion

What explains the stark price differences between the two individual plans offered by BCBS? First, as shown in Supporting Information S1: Table A6, there were no significant changes in the characteristics of the hospital market in Arkansas during the study period—such as hospital sizes and market concentration—that could have affected hospital–insurer negotiations. On the insurer side, however, two main mechanisms potentially explain the price differences between BCBS's individual plans. The first is that the price discrepancy stems from differences in plan features and regulations between BCBS's ACA and existing individual plans, which may influence price negotiations despite both being individual plans. The second mechanism is tied to the state's unique Medicaid expansion, which resulted in increased BCBS individual plan enrollment (as discussed in Section 4.1). This expansion may have strengthened BCBS's bargaining leverage, enabling it to negotiate lower hospital prices—primarily for its ACA plans—compared to pre‐ACA individual plan prices.

While it is unknown whether BCBS negotiates rates collectively or separately for ACA and existing individual plans, securing price reductions exclusively for ACA plans under the second mechanism is institutionally plausible for two reasons. First, negotiated prices are typically locked in by contracts lasting two to 5 years, which may explain why existing plan prices remained largely unchanged after the ACA.^17^ These contractual frictions may have limited BCBS's ability to adjust prices for existing plans, incentivizing the insurer to negotiate lower prices only for its new ACA individual plans, which were not subject to such constraints. Second, as discussed in Section 2, BCBS stopped enrolling new members in its existing individual plans following the introduction of ACA plans. Moreover, enrollment in existing plans declined as many enrollees switched to ACA plans after the ACA rollout (Figure 1b). Therefore, from a strategic standpoint, securing a price discount exclusively for ACA plans may have been more advantageous for BCBS than pursuing smaller discounts for both types of individual plans.

In the following section, I investigate which of the two insurer‐side mechanisms better explains the price changes in BCBS's individual plans.

## 5 Potential Mechanisms

### 5.1 Differences in Plan Features and Regulations

One explanation for the stark price differences between BCBS's ACA and existing individual plan prices is that the two plans have different features crucial for price negotiations, including managed care type and provider network. Different managed care types have varying cost‐sharing and referral structures, affecting how patients are steered toward in‐network providers. Price negotiations also center on provider networks, which determine both hospital participation and reimbursement rates for in‐network providers.

However, in these key aspects, BCBS's ACA and existing individual plans are highly similar. Both are PPO plans and Table 4 shows that their hospital networks are highly comparable. According to Arkansas APCD hospital claims, 91% of BCBS individual plan claims originate from hospitals in‐network for both plans. No hospital is in‐network only for existing plans, and while 9% of claims come from hospitals in‐network only for ACA plans, this is likely due to fewer existing plan claims. Even if this indicates BCBS's ACA network is broader, larger networks typically raise, not lower, negotiated prices (Ho and Lee 2017).

**TABLE 4:** Hospital network comparison between BCBS ACA and existing individual plans.

	Only in ACA plan (2014 and 2015)	Only in existing plan (2013)	In both plans
Number of in‐network hospitals	34	0	40
Share of claims	8.9%	0%	91.1%

*Note:* The share of claims represents the proportion of BCBS individual claims—existing individual plans during the pre‐ACA period and ACA individual plans in the post‐ACA period—associated with the corresponding group of hospitals.

Another possible explanation is that the ACA's new provisions on risk pooling and premium pricing for individual plans may have incentivized BCBS to reduce costs. Specifically, the ACA prohibited insurers from denying coverage for pre‐existing conditions (“guaranteed issue”) and mandated coverage of essential health benefits, potentially increasing insurers' risk exposure and prompting premium hikes. However, the ACA also imposed stricter premium‐setting regulations, such as capping age‐based rate variations and imposing the “medical loss ratio rule” requiring insurers to allocate at least 80% of premium revenues to medical claims. These constraints may have prompted carriers to adopt cost‐reduction strategies, such as negotiating lower reimbursement rates, rather than raising premiums.

However, ACA regulations alone are unlikely to be the main reason for BCBS's price changes. First, the ACA also introduced risk‐mitigating policies like the “individual mandate,” requiring nearly all individuals to obtain coverage. This policy, in effect during the study period, aimed to lower risk levels. Moreover, the ACA's premium and CSR subsidies for exchange plans may have attracted healthier individuals into the risk pool (Eibner and Saltzman 2014).

More importantly, while some ACA provisions may have encouraged cost‐cutting by insurers, they did not inherently strengthen insurers' bargaining positions. As the bargaining model in Supporting Information S1: Section A1 suggests, providers agree to lower prices when their negotiation surplus increases—such as through higher patient volumes from larger insurers. However, the ACA provisions did not directly increase providers' surplus, as they primarily influenced insurers' risk pools and premium pricing.

If ACA regulations—without increasing hospitals' negotiation surplus—were the main driver of BCBS's lower prices, then hospitals with greater market power would likely have secured smaller price reductions due to their stronger negotiating positions. To test this, I estimate a regression model similar to Equation (1), interacting ACA_Ind with two proxies for hospital market power: being a large hospital (at or above the 75th percentile in bed counts) (Large_Hosp) and being a member of a hospital system (System) (Gaynor and Town 2012b). This analysis excludes existing individual plan claims during post‐ACA years, as Table 3 shows minimal year‐over‐year price changes for those plans. The estimation results in Table 5 indicate that the price difference between existing and ACA individual plans is not significantly smaller for large and system‐affiliated hospitals, contradicting the hypothesis.

**TABLE 5:** Estimation results: Changes in negotiated prices for large hospitals and system‐affiliates.

	Dependent variable: log(Negotiated price)	
	Large hospitals	System‐affiliated hospitals
	(1)	(2)
ACA_Ind	−0.176***	−0.144***
	(0.046)	(0.037)
Large_Hosp	0.069	
	(0.053)	
ACA_Ind × Large_Hosp	−0.010	
	(0.054)	
System		−0.097**
		(0.047)
ACA_Ind ×System		−0.067
		(0.047)
log(LOS)	0.153***	0.153***
	(0.019)	(0.019)
Year2014	−0.010***	−0.010***
	(0.004)	(0.004)
Constant	8.739***	8.708***
	(0.051)	(0.050)
DRG FE	X	X
Hospital FE	X	X
Observations	31,360	31,360
*R*‐squared	0.915	0.915
Number of hospitals	74	74

*Note:* Robust standard errors, clustered by hospitals, are presented in parentheses (***p <0.01 **p <0.05 *p <0.1). All specifications estimate a variant of Equation (1) using the set of inpatient claims for BCBS existing individual plans in the pre‐ACA period and ACA plan in the post‐ACA period (i.e., excluding post‐ACA existing plan claims). Thus, all specifications exlcudes Existing_Ind_Post. Column (1) interacts ACA_Ind with Large_Hosp, an indicator for large hospitals, defined as those with a bed count at or above the 75th percentile. Column (2) interacts ACA_Ind with System, an indicator for system‐affiliated hospitals. For rows “DRG FE” and “Hospital FE,” an X indicates that the corresponding fixed effects are included in the specification.

Finally, a related concern is that including Medicaid beneficiaries in Arkansas's ACA individual plans could have affected insurer–hospital price negotiations by altering the risk profile of the enrollee pool. However, as discussed earlier, it is important to note that the theoretical implications of risk exposure for insurers' bargaining positions are ambiguous. Moreover, there is little evidence that Arkansas ACA plans with Medicaid enrollees had higher risk levels than pre‐ACA individual plans. In fact, pre‐ACA individual plans often had high‐risk pools due to the absence of risk‐reducing regulations such as the ACA's individual mandate (Adler and Ginsburg 2016). Supporting Information S1: Table A7 in the Online Appendix supports this, showing that BCBS's existing individual plan enrollees were older than ACA plan enrollees, and that patients in existing plans tended to have higher DRG weights (indicating greater severity) than those in ACA plans.

### 5.2 Bargaining Leverage Gain Due To Size Increase

The second potential mechanism that may explain the price difference between the ACA and existing individual plans is an increase in BCBS's bargaining leverage resulting from ACA‐induced enrollment growth. In this section, I conduct additional analyses to assess how much of the observed price changes can be attributed to this mechanism. If increased bargaining leverage is the primary driver, BCBS would be expected to secure larger price reductions when its enrollment growth leads the counterpart hospital to gain a larger surplus from negotiation—as predicted by the bargaining model in Supporting Information S1: Section A1. The goal of this section is to evaluate whether there is empirical evidence consistent with this theoretical prediction.

#### 5.2.1 Share of Eligible Population

**FIGURE 3:**
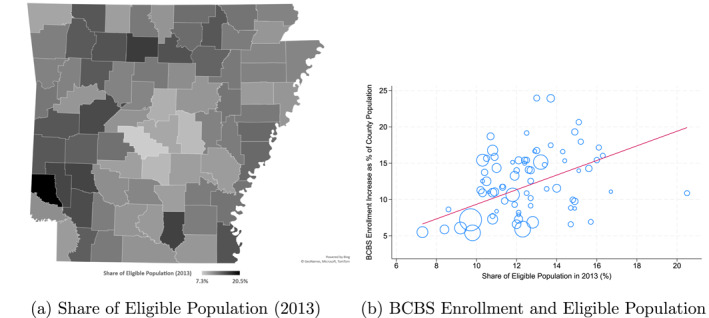
Share of the population eligible for aca medicaid expansion. *Source:* Author's calculation using the enrollment data from the arkansas APCD and small area health insurance estimates (SAHIE) data. The eligible population is defined as the non‐elderly adults (19–64 years old) whose household income level was lower than 138% of the FPL and who were uninsured in 2013. In figure (b), the vertical axis presents the BCBS enrollment increase between 2013 and 2015 as a share of the 2015 non‐elderly adult population at the county level. Additionally, the observations in figure (b) are weighted by the 2015 county‐level total non‐elderly adult population, with circle size representing the weights.

If the ACA‐induced enrollment increase for BCBS is the primary mechanism behind the price reduction for its individual plans, BCBS would be expected to secure larger price reductions at hospitals located in areas with greater growth in individual plan enrollment, as such growth generates a larger negotiation surplus for those hospitals. I assess this possibility by examining BCBS's price reductions across counties within Arkansas with differing degrees of enrollment increase. Specifically, I use the 2013 county‐level share of the population eligible for the ACA Medicaid expansion (hereafter, the share of the eligible population). An individual in 2013 is considered eligible if they were 19–64 years old, had a household income below 138% of the FPL, and were uninsured.^18^ Figure 3a shows the variation in this measure across Arkansas counties. Since Medicaid expansion was the primary driver of BCBS enrollment growth, as demonstrated in Figure 1, this measure effectively predicts actual changes in BCBS individual plan enrollment, as shown in Figure 3b.

I use the eligible population, which reflects the *expected* enrollment growth, rather than the actual growth of patient volume or enrollment, for two key reasons. First, because the eligible population is based solely on the geographic variation in pre‐ACA demographics, it is unlikely to be influenced by endogenous hospital choices. In contrast, actual changes in patient volume after the ACA may be shaped by hospitals' bargaining positions, which are themselves influenced by beneficiaries' hospital preferences. Second, this measure better aligns with the timing of negotiations, which occurred before enrollment for ACA individual plans began. CMS required carriers to submit their individual plan offerings by April 2013, while open enrollment did not begin until October 2013 (CMS 2013). This implies that plan features shaped by insurer–hospital negotiation—such as premiums and provider networks—had to be finalized before enrollment began. Therefore, anticipated enrollment changes—rather than actual ones—likely shaped the outcomes of these negotiations.

Using the eligible population measure, I assess whether variation in the anticipated enrollment increase explains the variation in negotiated price changes. Specifically, I estimate the following regression model, which is a variant of Equation (1):

(2) $$\begin{array}{l} \log\left(P_{\mathrm{ijdt}}\right)=\;\beta_{0}+\beta_{1}\;{ACA\text{\_}Ind}_{i}+\beta_{2}\;{Share}_{j}+\beta_{3}\;{ACA\text{\_}Ind}_{i}\times {Share}_{j}\\ \;+\beta_{l}\;\log\left({LOS}_{i}\right)+\delta_{d}+\alpha_{j}+\beta_{t}\;{Year2014}_{i}+\varepsilon_{\mathrm{ijdt}}. \end{array}$$

Similar to Table 5, this specification primarily compares prices between existing individual plans in the pre‐ACA period and ACA plans in the post‐ACA years, excluding post‐ACA existing plan claims. Share_j_ represents the share of the eligible population in 2013 in the county hospital j is located. Share_j_ is interacted with ACA_Ind_i_ to examine the relationship between the price change and the share of eligible population. Other variables are defined as in Equation (1). Data for Share_j_ are sourced from the Small Health Area Insurance Estimates published by the U.S. Census Bureau.

Table 6 reports the estimation results. Column (1) shows that the price drop for BCBS ACA plans is greater in counties with higher shares of the eligible population. This result indicates that a one‐percentage‐point increase in the share of the eligible population is associated with approximately a 2.3 log points (2.3%) lower price for the ACA plan than the existing plan, although its statistical significance is marginal. This result is consistent with the theoretical expectation that BCBS enjoys a greater bargaining advantage in areas with larger anticipated enrollment increases.

**TABLE 6:** Estimation results: Role of eligible population shares in price changes.

	Dependent variable: log(Negotiated price)	
	Entire claim set	Without the largest system
	(1)	(2)
ACA_Ind	0.073	0.192
	(0.158)	(0.158)
Share	−0.277***	−0.272***
	(0.030)	(0.031)
ACA_Ind ×Share	−0.023*	−0.033**
	(0.014)	(0.014)
log(LOS)	0.153***	0.160***
	(0.019)	(0.020)
Year2014	−0.010***	−0.010***
	(0.004)	(0.004)
Constant	12.221***	12.128***
	(0.360)	(0.378)
DRG FE	X	X
Hospital FE	X	X
Observations	31,360	27,671
*R*‐squared	0.915	0.911
Number of hospitals	74	71

*Note:* Robust standard errors, clustered by hospitals, are presented in parentheses (***p <0.01 **p <0.05 *p <0.1). All specifications estimate Equation (2). Column (1) includes all BCBS individual plan claims incurred in Arkansas hospitals, while Column (2) excludes the hospitals that are member hospitals of the state's largest hospital system and located in Pulaski County. For rows “DRG FE” and “Hospital FE,” an X indicates that the corresponding fixed effects are included in the specification.

However, an alternative explanation for the result is that the observed relationship between the price reduction and the share of the eligible population is driven by a few select hospitals in a single county. Several member hospitals of the largest hospital system in Arkansas are located in Pulaski County—the state's most populous county—which has the second‐lowest share of the eligible population. Because large hospital systems are generally understood to have a bargaining advantage (Gaynor and Town 2012b), the smaller price decrease in Pulaski County may be attributed not to its smaller share of the eligible population but to the market power of the hospitals affiliated with the largest system.

Column (2) in Table 6 tests this possibility by excluding the two hospitals in Pulaski County that are members of the largest hospital system. The estimation results show that the negative relationship between the eligible population and price reduction is strengthened when those two hospitals are excluded. A one‐percentage‐point increase in the share of the eligible population is associated with approximately a 3.3 log points (3.2%) lower price for the ACA plan. This suggests that the member hospitals of the largest hospital system are not the primary contributors to the negative relationship. Supporting Information S1: Table A3 shows that the estimation results in Table 6 are qualitatively robust when using a balanced set of hospitals with matched DRGs, as in Column (2) of Table 3, when including logged DRG weights as a control variable, and when applying the GLM specification.

#### 5.2.2 Service Volumes

The extent to which BCBS's enrollment increase grants it a bargaining advantage in negotiations with hospitals varies not only across geographic regions but also across types of hospital services. This variation arises because utilization levels differ by service, and prices are negotiated separately across services—even within the same insurer–hospital negotiation (White and Whaley 2019; Cooper, Craig, Gaynor, and Van Reenen 2019). As such, if the enrollment growth is the primary mechanism enhancing BCBS's bargaining leverage, its negotiated prices are expected to be particularly lower for services highly utilized by its ACA individual plan enrollees—that is, services for which hospitals' surplus is expected to increase more.

**FIGURE 4:**
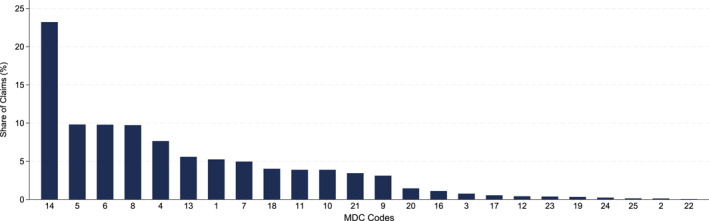
Percent frequencies of major diagnostic categories (MDC). *Source:* Author's calculation using the inpatient claims data from the arkansas APCD. The 2014 crosswalk maintained by the national bureau of economic research is used to map DRG codes to MDC codes. This figure shows the percent frequencies of MDC codes among the inpatient claims for the ACA medicaid beneficiaries enrolled in the BCBS plan in 2014 and 2015. There is no claim with MDC code 15 in the data.

To determine whether this hypothesis holds, I examine the variation in BCBS's price reductions for its individual plans across major diagnostic categories (MDC), a broader DRG‐based classification system. Using the 2014 crosswalk from the National Bureau of Economic Research, I map DRG codes to 25 MDC codes. Since newly insured ACA Medicaid beneficiaries largely drove BCBS's enrollment increase, the number of claims for the services most frequently used by these enrollees likely increased the most. Thus, I use the share of MDC codes among BCBS claims for Medicaid beneficiaries to determine the frequency of service usage. I assume that MDC frequency distribution is exogenous to negotiated prices, as patients typically lack awareness of negotiated prices before receiving care (GAO 2011). Figure 4 shows the percent frequencies of MDC codes among inpatient claims for Medicaid beneficiaries in BCBS's ACA individual plans. Supporting Information S1: Table A8 provides the full list of MDC codes, including descriptions, numbers of DRG codes, and percent frequencies.

To determine whether BCBS receives greater price discounts for the services with higher volumes of ACA Medicaid patients, I estimate the following regression model, which is another variant of Equation (1):

(3) $$\begin{array}{l} \log\left(P_{\mathrm{ijdt}}\right)=\;\beta_{0}+\beta_{1}\;{ACA\text{\_}Ind}_{i}+\beta_{2}\;\log\left({MDC\text{\_}Share}_{d}\right)+\beta_{3}\;{ACA\text{\_}Ind}_{i}\times \log\left({MDC\text{\_}Share}_{d}\right)\\ \;+\beta_{l}\;\log\left({LOS}_{i}\right)+\delta_{d}+\alpha_{j}+\beta_{t}\;{Year2014}_{i}+\varepsilon_{i} \end{array}$$

MDC_Share_d_ refers to the percent frequency of the MDC code to which the claim's DRG code d belongs. All other variables are defined in the same way as in Equation (1). As the distribution of the percent frequency of MDC codes is right‐skewed, I use the logged percent frequency of MDC codes in the model. I estimate the model using the same set of BCBS individual plan claims as in Section 5.2.1.

Table 7 reports the estimation results. Column (1) shows that services with higher patient volumes exhibit a greater price difference between BCBS's ACA and existing individual plans. A 1% increase in MDC_Share is associated with a 3.7 log point (3.6%) decrease in the price difference. Column (2) shows that the results are similar when using the balanced set of hospitals with matched DRGs. These results support the conceptual expectation that an insurer with increased enrollment has an incentive to negotiate larger discounts for the services used in higher volume. Thus, consistent with the results in Section 5.2.1, these findings highlight the importance of the insurer's bargaining position—shaped by its increase in size—in determining negotiated hospital prices.

**TABLE 7:** Estimation results: Role of service volumes in the price changes.

	Entire claim set	Balanced hospitals and DRGs
	(1)	(2)
ACA_Ind	−0.280***	−0.284***
	(0.030)	(0.034)
MDC_Share	0.457***	0.446***
	(0.025)	(0.008)
ACA_Ind × MDC_Share	−0.037***	−0.037***
	(0.009)	(0.012)
log(LOS)	0.152***	0.092***
	(0.019)	(0.019)
Year2014	−0.011***	−0.002
	(0.004)	(0.004)
Constant	11.456***	11.555***
	(0.108)	(0.070)
DRG FE	X	X
Hospital FE	X	X
Observations	31,154	13,707
*R*‐squared	0.914	0.945
Number of hospitals	74	40

*Note:* Robust standard errors, clustered by hospitals, are presented in parentheses (***p <0.01 **p <0.05 *p <0.1). All specifications estimate Equation (3). Column (1) uses the full set of BCBS individual plan claims, while Column (2) focuses on the same combinations of DRG codes and hospitals as those observed in pre‐ACA existing individual plan claims. For rows “DRG FE” and “Hospital FE,” an *X* indicates that the corresponding fixed effects are included in the specification.

The results remain robust when MDC group indicators are used instead of log(MDC_Share) to capture the variation in service volume. Column (1) of Supporting Information S1: Table A4 reports the estimation results from an alternative specification in which MDC group indicators—classifying codes into four categories based on their relative frequency ranks—are interacted with ACA_Ind. The results show that the group of most common services has the largest price reduction, whereas the group of least common services has the smallest price reduction. Supporting Information S1: Table A4 also demonstrates that the results remain robust when including log(DRGW) instead of DRG fixed effects (Column (2)). This rules out the alternative explanation that the results in Table 7 are influenced by the smaller number of claims per DRG code for less common services compared to more common services. Finally, Column (3) of Supporting Information S1: Table A4 shows that the results are robust when applying the GLM model specification in the estimation.

#### 5.2.3 Out‐of‐State Hospitals

Since BCBS's size increase was primarily driven by Arkansas' unique Medicaid expansion, which utilized ACA individual plans, this growth is not expected to increase the surplus from negotiations for non‐Arkansas hospitals, whose primary patient base consists of residents from their respective states. To further assess the role of insurer size in BCBS's price changes, Section A3 in the Supporting Information S1 examines how BCBS's prices changed for two in‐network hospitals in a neighboring state after the ACA. These hospitals are located outside Arkansas but had a reasonable number of claims for BCBS individual plans in the Arkansas APCD data both before and after the ACA. Notably, despite being out‐of‐state, these hospitals are in‐network for BCBS individual plans, meaning BCBS negotiated their reimbursement rates for individual plan beneficiaries.

In this analysis, I estimate the same regression specification as in Equation (1) using out‐of‐state hospital claims. Since I consider only two hospitals, I estimate the model separately for each. The estimation results reported in Supporting Information S1: Table A1 in the Online Appendix show that, unlike in‐state hospital prices, ACA plan prices for these out‐of‐state hospitals did not decrease relative to pre‐ACA individual plan prices. In fact, for one of the two hospitals, ACA plan prices were higher than pre‐ACA individual plan prices. Supporting Information S1: Table A1 further examines a subset of Arkansas hospitals that share similar characteristics with the out‐of‐state hospitals, with DRG codes matched to those for out‐of‐state hospital claims. The results continue to indicate price reductions for BCBS ACA plans among in‐state hospitals, consistent with the overall findings in Table 3. Aligning with the results in Sections 5.2.1 and 5.2.2, the findings in Supporting Information S1: Table A1 underscore the crucial role of BCBS's enhanced bargaining position—resulting from its size increase—in driving hospital price variation.

## 6 Discussion: Magnitude and Implication of the Size Effect

The 16.7% reduction in negotiated hospital prices documented in Section 4.2 is substantial relative to both annual hospital price growth and prior studies.^19^ One possible reason is that most ACA plan enrollees in Arkansas were Medicaid beneficiaries. Medicaid reimbursement rates are typically lower than commercial and Medicare rates. While Arkansas‐specific data is unavailable, Selden et al. (2015) report that, on average, Medicaid inpatient rates were 90% of Medicare rates, whereas commercial plans paid 175.3% of Medicare rates in 2012. Thus, had Arkansas used traditional Medicaid or MMC plans instead of ACA individual plans, hospitals could have received even lower reimbursements for Medicaid patients. If hospitals considered this in negotiations, accepting lower prices for BCBS's ACA plans may have been a reasonable tradeoff—as long as prices remained above Medicaid rates. Clemens and Gottlieb (2016) show that public program rates can influence commercial plan prices.

Thus, despite its magnitude, the welfare implications of the size effect remain unclear. On the one hand, the price reduction translates into significant savings in hospital expenditures: a back‐of‐the‐envelope calculation from Section A4 in the Supporting Information S1 suggests that a 16.7% price reduction lowered BCBS's hospital expenditures by $119.94 per member per year, representing 14.6% of the state's per capita inpatient spending. Lower hospital costs, in turn, imply reduced premium payments for both non‐Medicaid plan beneficiaries and the Arkansas government, which subsidized premiums for ACA Medicaid beneficiaries. On the other hand, Medicaid spending could have been lower under traditional Medicaid or MMC, which generally involve lower reimbursement rates.

Furthermore, a full cost–benefit analysis must also consider the potential advantages ACA individual plans offered to Medicaid beneficiaries, such as broader provider networks and better access to care. Traditional Medicaid and MMC beneficiaries tend to face lower provider acceptance rates (Decker 2012; Polsky et al. 2015), and MMC plans often have narrower networks (Graves et al. 2020). Additionally, transitioning to an MMC model in Arkansas would have incurred extra administrative costs, as the state primarily relied on traditional Medicaid plans for its pre‐ACA Medicaid program. Therefore, assessing the full impact of the ACA‐induced size effect on expenditures and social welfare is beyond the scope of this paper.

## 7 Conclusion

This paper examines how insurer size affects hospital price negotiations in the United States. My analysis reveals that Arkansas BCBS, which experienced an enrollment increase due to the state's unique approach to Medicaid expansion, negotiated significantly lower hospital prices. Consistent with the bargaining model, my findings indicate that the Arkansas BCBS secured lower prices particularly when its size increase generated a greater surplus for hospitals from the negotiation.

These results have important implications for the growth of major U.S. commercial insurers, which have expanded nationally through diverse market segments, such as Medicare Advantage (Schoen and Collins 2017). My findings suggest that growing insurer size can influence provider negotiations and healthcare prices, even without a corresponding increase in market concentration. The results also have implications for future healthcare reforms. Proposals to expand health coverage in the United States often involve models of publicly subsidized private plans, similar to Arkansas's ACA Medicaid expansion model (Blumberg et al. 2019). My findings suggest that such models can affect private insurance plan prices and expenditures by influencing enrollments. Therefore, policymakers should consider these effects when designing future reforms, in addition to the reforms' impacts on healthcare beneficiaries and providers.

## Conflicts of Interest

The author declares no conflicts of interest.

## Supporting information

Supporting Information S1

## Data Availability

The data that support the findings of this study are available from Arkansas All‐Payer Claims Database. Restrictions apply to the availability of these data, which were used under license for this study. Data are available from the author(s) with the permission of Arkansas All‐Payer Claims Database.

## Endnotes

^1^
For example, see the following newspaper articles: Nunn (2018) and Davies (2018).

^2^
The following papers theoretically examine the role of buyer size in price negotiation: Galbraith (1952), Stole and Zwiebel (1996), Snyder (1996), Brooks et al. (1997), Chipty and Snyder (1999), and Raskovich (2003).

^3^
In the health economics literature, a provider's surplus from negotiation is often measured by its profit gain from being included in the insurer's provider network (Town and Vistnes 2001; Capps et al. 2003; Ho 2009; Lewis and Pflum 2015; Gowrisankaran et al. 2015).

^4^
Papers in this strand of literature include G. A. Melnick et al. (1992), Sorensen (2003), Wu (2009), Moriya et al. (2010), Ho and Lee (2017), Barrette et al. (2022), and LoSasso et al. (2023) for hospital services; Ellison and Snyder (2010) and Lakdawalla and Yin (2015) for pharmaceutical drugs; and E. T. Roberts et al. (2017) and Scheffler and Arnold (2017) for professional services.

^5^
As mentioned earlier, this literature includes G. A. Melnick et al. (1992), Sorensen (2003), Wu (2009), Ellison and Snyder (2010), Lakdawalla and Yin (2015), Ho and Lee (2017), E. T. Roberts et al. (2017), Scheffler and Arnold (2017), Ho and Lee (2019), Barrette et al. (2022), and LoSasso et al. (2023).

^6^
This research often focuses on healthcare markets. Comprehensive reviews of the impact of hospital market concentration and consolidation are provided by Gaynor and Town (2012a) and Gaynor et al. (2015). More recent studies include Gowrisankaran et al. (2015), Garmon (2017), and Lewis and Pflum (2017). Beyond healthcare, studies like Chipty (1995), Chipty and Snyder (1999), Crawford and Yurukoglu (2012), and Zhang and Chung (2020) explore the role of firm size in upstream negotiations in industries such as cable TV and online platforms.

^7^
For example, Goudie et al. (2020) and Bollinger et al. (2021) find higher utilization and greater satisfaction among Medicaid beneficiaries enrolled in individual plans compared to traditional Medicaid enrollees. In contrast, Sommers et al. (2016), (2017) show that changes in healthcare utilization, access to care, and health outcomes among ACA Medicaid beneficiaries in Arkansas are comparable to those observed in Kentucky, which primarily used MMC plans for its ACA Medicaid expansion.

^8^
The ACA established tiers for exchange plans based on their actuarial values: Bronze, Silver, Gold, and Platinum. The premium and cost‐sharing subsidies are not available for off‐exchange plan enrollees. For more information about the ACA's regulations and provisions for ACA individual plans, refer to “Summary of the Affordable Care Act,” published in 2013 by the Kaiser Family Foundation (https://www.kff.org/affordable‐care‐act/fact‐sheet/summary‐of‐the‐affordable‐care‐act/).

^9^
Arkansas's ACA Medicaid program underwent additional major changes, including the introduction of a work requirement in 2018; however, these changes occurred after the study period of this paper.

^10^
While comparing individual procedures could be an alternative approach, greater variability in procedures, the potential for bundling, and the lack of internally assigned classifications—such as DRG codes for inpatient admissions—make it more difficult to compare overall hospital prices using this method.

^11^
Outliers are defined as claims that meet at least one of the following conditions: (1) the DRG weight is greater than the 99th percentile; or (2) the negotiated amount is either greater than the 99th percentile or less than the 1st percentile.

^12^
Two hospitals in Texarkana, Texas, that appear in the data are coded as Arkansas hospitals because Texarkana is a twin city spanning both Texas and Arkansas.

^13^
Enrollment and patient data are sourced from Health Insurance & Managed Care Indicators by the KFF. Data on the non‐elderly adult population are from the Annual Estimates of the Resident Population for Selected Age Groups by Sex for Arkansas: April 1, 2010, to July 1, 2019, published by the U.S. Census Bureau.

^14^
This statistic is from the 2014 monthly enrollment report by the Arkansas Department of Human Services. For more details, see Ramsey (2014).

^15^
In Supporting Information S1: Section A2, I discuss the potential implications if the assumption that BCBS's growth was largely exogenous to price negotiations does not hold.

^16^
Additional analysis, not reported in the paper, confirms that negotiated hospital prices do not systematically differ between Medicaid and non‐Medicaid enrollees in BCBS ACA individual plans.

^17^
Dunn and Shapiro (2015) and Cooper, Craig, Gaynor, and Van Reenen (2019) discuss this practice, which was also confirmed through an interview with a hospital system professional in Arkansas.

^18^
This approach follows Courtemanche et al. (2019) and Dunn et al. (2021), who use similar measures to assess county‐level exposure to the ACA Medicaid expansion.

^19^
According to Cooper, Craig, Gaynor, Harish, et al. (2019), hospital prices for inpatient services grew by 42% between 2007 and 2014, translating to 6% annual growth. Similarly, UnitedHealth (2019) report that hospital prices for inpatient services grew by 19% between 2013 and 2017, translating to 4.5% annual growth. Moreover, Lakdawalla and Yin (2015) find that the increase in insurer size due to the introduction of Medicare Part D (which covers pharmaceutical drugs via private plans) lowered pharmacy prices by an average of 3.7%.
