# Supplementary material for: Insurer Size and Negotiated Hospital Prices: Insights From the Affordable Care Act in Arkansas
Source: Health Econ. 2025 Aug 1;34(11):2097–113. doi: 10.1002/hec.70022 (PMC12496023; doi:10.1002/hec.70022)
Supplement: Supplementary file 1 — Supporting Information S1 [file HEC-34-2097-s001.pdf]

Online Appendix to "Insurer Size and Negotiated Hospital Prices: Insights from the Affordable Care Act in Arkansas"*

Jee-Hun Choi†

Lehigh University

*The findings presented represent an independent analysis conducted by the author and do not represent the opinions of the Arkansas Insurance Department or the Arkansas Healthcare Transparency Initiative. Approval to use data from the Arkansas All-Payer Claims Database for this analysis is not an endorsement of the results presented herein. All errors are my own.

†Lehigh University, Rauch Business Center 476, 621 Taylor Street, Bethlehem, PA 18015, jec720@lehigh.edu.

A1 Theoretical Framework

In this section, I use a stylized theoretical model to delineate how an insurer size increase may affect bilateral price negotiation between insurers (managed care plans) and hospitals.[1] Specifically, I use a Nash bargaining model between an insurer and a hospital in which they determine the price the insurer pays to the hospital (Lewis and Pflum, 2015; Gowrisankaran, Nevo and Town, 2015; Ho, 2009).[2] In line with the extant literature, I make the following three assumptions in the model. First, in each bilateral negotiation, an insurer and a hospital maximize the product of their profit surpluses generated by including the hospital in the insurer's hospital network (i.e., Nash product) (Crémer and Riordan, 1987). Second, in each negotiation, both parties take the results of other negotiations as given, and each negotiation is not affected by the results of other negotiations (Horn and Wolinsky, 1988). Third, when an insurer and a hospital do not reach an agreement in their negotiations, the hospital is excluded from the insurer's network, and the beneficiaries of the insurer switch to alternative hospitals in the hospital network.

Let $\Pi_m(\mathcal{M})$ be the profit function of insurer m. It is defined as the difference between beneficiaries' willingness-to-pay for their hospital network, $\mathcal{M}$ (i.e., beneficiaries' expected utility from $\mathcal{M}$), and hospital payments.[3] Then, $\Delta_h\Pi_m$, the surplus to insurer m as a result of including hospital h in its network, is given as $\Pi_m(\mathcal{M}) - \Pi_m(\mathcal{M}\backslash h)$. Alternatively, let $\Pi_h(\mathcal{H})$ be hospital h's profit. It is defined as the difference between the total reimbursements received and the costs to treat the patients from $\mathcal{H}$, the group of insurers having hospital h in their hospital networks. Then, the surplus to hospital h as a result of being included in insurer m's hospital network, $\Delta_m\Pi_h$, is given as $\Pi_h(\mathcal{H}) - \Pi_h(\mathcal{H}\backslash m)$.

Based on the assumptions made above for the bargaining game, the insurer's and hospital's surpluses can be expressed like below (using the notations of Lewis and Pflum, 2015):

$$\Delta_h\Pi_m = \Delta_h W_m(\mathcal{M}) - \Big(\sum_{k\in\mathcal{M}} \mathbf{p}_{km} D_k(\mathcal{M}) - \sum_{k\in\mathcal{M}\backslash h} \mathbf{p}_{km} D_k(\mathcal{M}\backslash h)\Big), \tag{A1}$$

$$\Delta_m\Pi_h = \mathbf{p}_{hm} D_h(\mathcal{M}) - \Delta_m C_h(D_h(\mathcal{M})), \tag{A2}$$

[1] A managed care plan contracts with medical providers (such as hospital systems and physician groups) at a local level to include the member providers in its provider network. A provider network for a managed care plan is the group of medical providers that the plan members can use for healthcare services. Although managed care plans cover most of the services performed by in-network providers, they do not cover—or only partially cover—the services performed by providers that do not belong to the network (i.e., "out-of-network providers"). This feature contrasts with that of traditional fee-for-service plans, which cover the services rendered by any provider.

[2] This model can be generalized to the negotiations between an insurer and a hospital system.

[3] I follow the literature for the definition of insurer profit (e.g., Capps, Dranove and Satterthwaite, 2003; Lewis and Pflum, 2015; Gowrisankaran, Nevo and Town, 2015).

where $\Delta_h W_m(\mathcal{M})$ is the change in beneficiaries' willingness-to-pay when hospital h joins insurer m's network; $\mathbf{p}_{km}$ is the negotiated price between hospital k and insurer m; $D_k(\mathcal{M})$ is beneficiaries' demand for hospital k from insurer m's network $\mathcal{M}$; and $\Delta_m C_h(D_h(\mathcal{M}))$ is the change in hospital h's cost when it joins insurer m's network.

The objective function in this bargaining game can be written as

$$\max_{\mathbf{p}_{hm}} [\underbrace{\Pi_m(\mathcal{M}) - \Pi_m(\mathcal{M}\backslash h)}_{\Delta_h \Pi_m}]^{1-\alpha_h} [\underbrace{\Pi_h(\mathcal{H}) - \Pi_h(\mathcal{H}\backslash m)}_{\Delta_m \Pi_h}]^{\alpha_h}, \tag{A3}$$

where $\alpha_h \in [0, 1]$. Then, using the definitions of the surpluses above, the first-order condition of Equation (A3) with respect to $\mathbf{p}_{hm}$ yields the following equilibrium bargaining outcome:

$$\Delta_m \Pi_h(\mathbf{p}_{hm}) = \alpha_h \left[\Delta_m \Pi_h(\mathbf{p}_{hm}) + \Delta_h \Pi_m(\mathbf{p}_{hm})\right]. \tag{A4}$$

Assuming that α_h and $\Delta_m \Pi_h(\mathbf{p}_{hm})$ are nonzero, Equation (A4) can be written as

$$\frac{1}{\alpha_h} = 1 + \frac{\Delta_h \Pi_m(\mathbf{p}_{hm})}{\Delta_m \Pi_h(\mathbf{p}_{hm})}. \tag{A5}$$

Equation (A5) implies that the equilibrium bargaining outcome is influenced by two factors: (1) the ratio between the two parties' surpluses generated in the negotiation ($\Delta_h \Pi_m(\mathbf{p}_{hm})$ $/\Delta_m \Pi_h(\mathbf{p}_{hm})$) and (2) how much of the joint surplus is split between the two parties (α_h). The extant health industrial organization literature refers to the first factor as *bargaining leverage*, which describes how the potential surpluses of the two parties influence the negotiated prices. The latter factor, α_h (referred to as *bargaining power* in the literature), is often used as a term capturing the administrative factors beyond bargaining leverage that may affect price negotiation, such as bargaining skills and resources.

Equation (A5) implies that the size or market share of each party is a crucial factor in the price negotiation. For example, suppose a hospital negotiates prices with two insurers in the same market, one with relatively larger market share than the other. All else being equal, the hospital's surplus from negotiation, $\Delta_m \Pi_h$, is larger when successfully negotiating with the larger insurer than the smaller insurer because the hospital loses a larger pool of patients if negotiations fail with the larger insurer. Then, with hospital's surplus, $\Delta_h \Pi_m$, unchanged, the equilibrium condition in Equation (A5) exerts greater downward pressure on $\mathbf{p}_{hm}$ for the larger insurer than for the smaller one. As a result, the equilibrium negotiated price, $\mathbf{p}_{hm}$, will be lower for the larger insurer than the smaller insurer.

This framework can also be used to predict how an increase in an insurer's enrollment may affect their price negotiation. If the enrollment of an insurer rises while all other factors

remain unchanged, the number of patients covered by the insurer also grows. Consequently, the hospital's surplus, $\Delta_m \Pi_h$, increases because, although its profit in the event of disagreement ($\Pi_h(\mathcal{H} \backslash m)$) remains unchanged, its profit when reaching an agreement ($\Pi_h(\mathcal{H})$) is now larger. On the other hand, the change in the insurer's surplus, $\Delta_h \Pi_m$, is not as large because the insurer faces no shift in its enrollees' hospital preferences or in the broader hospital market structure. Therefore, the increase in $\Delta_m \Pi_h(\mathbf{p}_{hm})$ resulting from the insurer's expanded size puts downward pressure on $\mathbf{p}_{hm}$ to satisfy the equilibrium condition in Equation (A5). Consequently, an insurer with higher enrollment is expected to have greater bargaining leverage, allowing it to negotiate lower prices.[4]

A2 Endogeneity in Plan Choices

In my analyses in Section 5.2 of the main manuscript, I assume that the enrollment increase of the BCBS individual plans, spurred by the ACA, is plausibly exogenous to price negotiation. This assumption is based on three reasons, detailed in Section 4.1. First, in some local markets, BCBS was the only insurer offering exchange plans. Second, over half of the Medicaid beneficiaries, the main source of size increase, did not self-select their plans. Lastly, the financial attributes of ACA individual plans are unlikely to affect the plan choices of Medicaid beneficiaries, who constituted approximately 80% of BCBS's ACA individual plan beneficiaries, as they did not pay any premiums. However, this does not imply that plan choices are entirely exogenous. There remains a possibility that my empirical results are influenced to some extent by endogeneity in plan choices. Therefore, in this section, I discuss how such endogeneity in plan choices can impact my empirical findings.

Conceptually, the selection of BCBS exchange plans by Arkansas Medicaid beneficiaries over other options could be driven by two types of preferences. First, Medicaid beneficiaries may choose BCBS exchange plans because they favor BCBS over other insurance carriers. This preference could arise from its nationally recognized brand name or beneficiaries' prior experiences with it, given that BCBS has a long-standing presence in the state, offering both individual and group plans. In this scenario, endogenous plan choices would likely provide a bargaining advantage to BCBS; beneficiaries opting for BCBS over other insurers

[4] The particular Nash bargaining game used in this section is not the only theoretical model predicting the impact of buyer size on price negotiation. Prior studies in the theoretical literature have shown the existence of the buyer-size effect using different models in various market settings (Stole and Zwiebel, 1996, Snyder, 1996, Brooks, Dor and Wong, 1997, Chipty and Snyder, 1999, Raskovich, 2003). They show that given the concavity of the supplier's surplus function—due to a capacity constraint or a convex cost function—a larger buyer can negotiate lower prices. In other words, similarly to the implication of Equation (5) in the Nash bargaining model, larger buyers have bargaining advantage by generating greater per-unit surpluses for suppliers (i.e., bargaining leverage is increased).

are unlikely to switch plans even if a provider is removed from BCBS's network.

Alternatively, Medicaid beneficiaries can be drawn to BCBS plans because of a strong preference for specific providers accessible through the plan's broad provider network. In this scenario, endogenous plan choices would not necessarily offer a bargaining advantage to BCBS. Beneficiaries who choose BCBS due to preferred providers might consider switching to a different plan if those providers leave BCBS's network.

Consequently, the influence of endogenous plan choices on price negotiations between BCBS and hospitals depends on which preference between the two is more prevalent. If Medicaid beneficiaries mainly choose BCBS plans due to a preference for the insurer itself, the price effect of a size increase outlined in Section 4.2 could be overestimated, depending on the degree of endogeneity in plan choices. This is because the increase in BCBS's bargaining leverage partly results from endogenous plan choice. On the other hand, if beneficiaries predominantly select BCBS based on specific provider preferences, the price effect described in Section 4.2 could be underestimated, with the extent dependent on the endogeneity of these choices.

Given that the majority of BCBS exchange plan beneficiaries are newly insured Medicaid beneficiaries who recently obtained insurance coverage through the state's Medicaid expansion, it is unlikely that many of them possess a strong preference for specific providers. Therefore, there is a greater possibility that endogenous plan choices are influenced more by a preference for BCBS than by a preference for providers. This could provide another explanation for the observed larger price effect compared to earlier studies, discussed in Section 6. However, pinpointing which preference predominates in my setting lies beyond the scope of this paper due to limited variation in plan choices and a lack of information about exactly which Medicaid beneficiaries self-selected BCBS plans.

A3 Out-of-State Hospitals

In this section, I examine how BCBS's ACA individual plan prices compare to its existing individual plan prices at out-of-state hospitals. I consider two hospitals located near the Arkansas state border, where claims for BCBS individual plans appear in reasonable numbers in the Arkansas APCD data. These cases primarily involve Arkansas residents living near the state border who sought care at hospitals in neighboring states. It is important to highlight that, although these are out-of-state hospitals, they are in-network for BCBS individual plans, meaning that BCBS still negotiated their reimbursement rates for individual plan

beneficiaries.[5]

Specifically, I re-estimate the regression specification in Equation (1) using out-of-state hospital claims. However, since this analysis includes only two hospitals, I estimate the equation separately for each, incorporating the following modifications. I cluster standard errors at the DRG level, and the specification excludes the 2014 indicator, as it may absorb price variation for the hospital in the post-ACA period.

Columns (1) and (2) of Table A1 report the estimation results. In contrast to in-state hospital prices presented in Table 3, individual plan prices for out-of-state hospitals did not decrease. For the first out-of-state hospital (Column (1)), negotiated hospital prices even increased by approximately 23.6 log points relative to existing individual plan prices. For the second out-of-state hospital (Column (2)), negotiated prices for ACA individual plans were not significantly different from existing individual plan prices.

One caveat when comparing in-state and out-of-state hospitals is that the latter may differ in characteristics from the in-state hospitals considered in this study. The out-of-state hospitals in this study are generally larger than the in-state hospitals. The 2015 bed counts for the two out-of-state hospitals were 1,397 and 555, whereas the median bed count for in-state hospitals in 2015 was 76. Additionally, both out-of-state hospitals are system-affiliated.

However, as shown in Table 5 in Section 5.1, lower negotiated prices for ACA individual plans were also observed among large hospitals and system-affiliated hospitals in Arkansas. Columns (3) and (4) of Table A1 estimate Equation (1), focusing on large and system-affiliated hospitals, using claims with DRG codes matched to those from out-of-state hospital claims. Both estimations indicate price reductions for BCBS's ACA plans relative to existing individual plan prices, consistent with the overall results in Table 3. Aligning with the results in Sections 5.2.1 and 5.2.2, the difference in results between out-of-state and in-state hospitals suggests that the insurer's bargaining leverage depends on how its size increase influences counterpart hospitals' surplus from negotiation.

A4 Calculating the Impact of the Size Effect on Hospital Expenditure

According to Arkansas APCD data, the median per-claim hospital expenditure for BCBS's ACA individual plan enrollees between 2014 and 2015 was \$6,434. Assuming BCBS's hospital prices for its ACA plan would have matched its existing individual plan prices in the absence

[5] An alternative specification for this analysis would be a difference-in-differences regression model using the set of out-of-state hospitals as a control group. However, since the pre-ACA period includes only a single year, it is not feasible to test the parallel trends assumption in this setting.

Table A1: Estimation Results: Negotiated Prices for Out-of-State Hospitals

	log(Negotiated Price)			
	Out-of-State Hospitals		In-State Hospitals	
	Out-of-State Hospital 1	Out-of-State Hospital 2	Large Hospitals Matched DRGs	System Affiliates Matched DRGs
	(1)	(2)	(3)	(4)
ACA_Ind	0.232***	0.003	−0.218***	−0.240***
	(0.065)	(0.060)	(0.027)	(0.031)
Existing_Ind_Post	0.263***	0.080	−0.007	−0.006
	(0.094)	(0.081)	(0.016)	(0.014)
log(LOS)	0.150*	0.164***	0.086***	0.082***
	(0.081)	(0.054)	(0.027)	(0.015)
Year2014			−0.006	−0.006
			(0.004)	(0.005)
Constant	8.929***	9.156***	8.991***	8.978***
	(0.130)	(0.102)	(0.049)	(0.032)
DRG FE	X	X	X	X
Hospital FE	X	X	X	X
Observations	456	279	7,342	7,034
R-Squared	0.926	0.958	0.957	0.953
Number of Hospitals	1	1	17	35

Notes: Robust standard errors, clustered by hospitals, are presented in parentheses (*** p<0.01 ** p<0.05 * p<0.1). Columns (1) and (2) present the results of estimating Equation (1) separately using BCBS individual plan claims for two out-of-state hospitals. In these estimations, standard errors are clustered at the DRG level, and the 2014 indicator ($\texttt{Year}_{2014}$) is excluded. Column (3) estimates Equation (1) using the set of in-state large hospitals, defined as those with the number of beds at or above the 75th percentile. Column (4) uses the set of large in-state hospitals and specifically focuses on the same DRG codes as those presented in the out-of-state hospitals' claims for the existing BCBS individual plan. For rows "DRG FE" and "Hospital FE," an X indicates that the corresponding fixed effects are included in the specification.

of a size increase, the 16.7% price reduction corresponds to savings of $1,290 ($\$6,434/(1-0.167)-\$6,434$) per claim, $37.8 million in total (given 29,293 inpatient claims), and $119.94 per member per year (given 315,052 member-years) over two years. This represents 14.6% of per-capita hospital care expenditures in Arkansas, which amounted to $821.7 in 2016, according to the 2016 Health Care Cost and Utilization Report by the Health Care Cost Institute.[6]

[6] https://healthcostinstitute.org/annual-reports/2016-health-care-cost-and-utilization-report

A5 Robustness Checks

Table A2: Estimation Results: Changes in Negotiated Prices (Robustness Check)

	Specifications with log(DRGW)		GLM	
	Entire Claim Set	Balanced Hospitals and DRG Codes	Entire Claim Set	Balanced Hospitals and DRG Codes
	(1)	(2)	(3)	(4)
ACA_Ind	−0.185***	−0.174***	−0.204***	−0.211***
	(0.024)	(0.026)	(0.025)	(0.026)
Existing_Ind_Post	−0.005	0.008	−0.005	−0.007
	(0.009)	(0.010)	(0.010)	(0.008)
log(LOS)	0.123***	0.069***	0.166***	0.103***
	(0.014)	(0.013)	(0.016)	(0.016)
Year2014	−0.008**	−0.007**	−0.012***	−0.005
	(0.003)	(0.003)	(0.003)	(0.003)
log(DRGW)	0.972***	1.002***		
	(0.008)	(0.005)		
Constant	8.677***	8.779***	10.125***	9.906***
	(0.032)	(0.035)	(0.100)	(0.050)
DRG FE			X	X
Hospital FE	X	X	X	X
Observations	34,828	15,541	34,828	15,541
R-Squared	0.901	0.929		
Number of Hospitals	74	40	74	40

Notes: Robust standard errors, clustered by hospitals, are presented in parentheses (*** p<0.01 ** p<0.05 * p<0.1). Columns (1) and (2) estimate a variant of Equation (1) that includes log(DRGW) as a control variable instead of using DRG fixed effects. Columns (3) and (4) estimate the Generalized Linear Model (GLM) specification, where the dependent variable is the negotiated price, P_{ijdt}, using the same set of independent variables as in Equation (1). The GLM specification uses a gamma distribution with a log link function for the dependent variable. Columns (1) and (3) use the full set of BCBS individual plan claims, while Columns (2) and (4) focus on the same combinations of DRG codes and hospitals as those observed in existing individual plan claims. For rows "DRG FE" and "Hospital FE," an X indicates that the corresponding fixed effects are included in the specification.

Table A3: Estimation Results: Role of Eligible Population Shares in Price Changes (Robustness Check)

	Balanced Hospitals and DRGs		Specifications with log(DRGW)		GLM	
	Balanced Claim Set	Without the Largest System	Entire Claim Set	Without the Largest System	Entire Claim Set	Without the Largest System
	(1)	(2)	(3)	(4)	(5)	(6)
ACA_Ind	0.095	0.231	0.045	0.164	0.049	0.170
	(0.173)	(0.171)	(0.157)	(0.154)	(0.161)	(0.166)
Share	0.052***	0.062***	−0.261***	−0.252***	0.114	0.109
	(0.014)	(0.014)	(0.015)	(0.014)	(0.073)	(0.076)
ACA_Ind×Share	−0.026*	−0.037**	−0.021	−0.031**	−0.023*	−0.033**
	(0.015)	(0.015)	(0.014)	(0.013)	(0.014)	(0.014)
log(LOS)	0.094***	0.097***	0.121***	0.128***	0.161***	0.168***
	(0.019)	(0.023)	(0.014)	(0.016)	(0.016)	(0.018)
Year2014	−0.002	−0.004	−0.008**	−0.008*	−0.010***	−0.011***
	(0.004)	(0.005)	(0.004)	(0.004)	(0.004)	(0.004)
log(DRGW)			0.972***	0.969***		
			(0.009)	(0.010)		
Constant	8.206***	8.074***	11.964***	11.835***	8.773***	8.704***
	(0.161)	(0.162)	(0.169)	(0.157)	(0.885)	(0.922)
DRG FE	X	X			X	X
Hospital FE	X	X	X	X	X	X
Observations	13,755	11,411	31,360	27,671	31,360	27,671
R-Squared	0.945	0.944	0.907	0.902		
Number of Hospitals	40	38	74	71	74	71

Notes: Robust standard errors, clustered by hospitals, are presented in parentheses (*** p<0.01 ** p<0.05 * p<0.1). Columns (1) and (2) estimate Equation (2) focusing on the same combinations of DRG codes and hospitals as those observed in the existing individual plan claims. Columns (3) and (4) estimate a variant of Equation (2) that includes log(DRGW) as a control variable instead of using DRG fixed effects. Columns (5) and (6) estimate the Generalized Linear Model (GLM) specification, where the dependent variable is the negotiated price, P_{ijdt}, using the same set of independent variables as in Equation (2). The GLM specification uses a gamma distribution with a log link function for the dependent variable. Columns (1), (3), and (5) use the full set of Arkansas hospitals, while Columns (2), (4), and (6) exclude the hospitals that are member hospitals of the state's largest hospital system and located in Pulaski County. For rows "DRG FE" and "Hospital FE," an X indicates that the corresponding fixed effects are included in the specification.

Table A4: Estimation Results: Role of Service Volumes in the Price Changes (Robustness Check)

	Specification with MDC Group Indicators	Specification with log(DRGW)	GLM
	(1)	(2)	(3)
ACA_Ind	−0.128***	−0.237***	−0.308***
	(0.031)	(0.030)	(0.029)
log(MDC_Share)		0.031***	0.458***
		(0.008)	(0.023)
ACA_Ind×log(MDC_Share)		−0.018**	−0.039***
		(0.008)	(0.009)
GroupB	1.560***		
	(0.076)		
GroupC	−0.397***		
	(0.093)		
GroupD	−0.665***		
	(0.057)		
Post×GroupB	−0.064***		
	(0.018)		
Post×GroupC	−0.051**		
	(0.025)		
Post×GroupD	−0.114***		
	(0.026)		
log(LOS)	0.152***	0.123***	0.161***
	(0.019)	(0.015)	(0.017)
Year2014	−0.011***	−0.007*	−0.011***
	(0.004)	(0.004)	(0.004)
log(DRGW)		0.976***	
		(0.008)	
Constant	8.570***	8.772***	11.484***
	−0.072	(0.034)	(0.101)
DRG FE	X		X
Hospital FE	X	X	X
Observations	31,154	31,154	31,154
R-Squared	0.914	0.906	
Number of Hospitals	74	74	74

Notes: Robust standard errors, clustered by hospitals, are presented in parentheses (*** p<0.01 ** p<0.05 * p<0.1). Column (1) estimates the specification that uses MDC group indicators instead of log(MDCShare): $\log(\mathtt{P}_{ijdt}) = \beta_0 + \beta_1\,\mathtt{ACA\_Ind}_i + \sum_M \beta_2^M\,\mathtt{GroupM}_d + \sum_M \beta_3^M\,\mathtt{ACA\_Ind}_i \times \mathtt{GroupM}_d + \beta_l \log(\mathtt{LOS}_i) + \sum_d \beta_d\,\mathtt{DRG}_d + \sum_j \alpha_j + \sum_t \beta_t\,\mathtt{Year}_t + \varepsilon_i$. $\mathtt{GroupM}_d$ takes a value of one if the MDC code corresponding to the claim's DRG code d belongs to Group M, where M $\in \{A, B, C, D\}$. The set of MDC codes is divided into four groups in the order of their frequencies, as shown in Table A8, with Group A being the least common and Group D being the most common. Each group accounts for approximately a quarter of the BCBS inpatient claims considered in the analysis. Column (2) estimates a variant of Equation (3) that includes log(DRGW) as a control variable instead of using DRG fixed effects. Columns (3) estimates the Generalized Linear Model (GLM) specification, where the dependent variable is the negotiated price, $\mathtt{P}_{ijdt}$, using the same set of independent variables as in Equation (3). The GLM specification uses a gamma distribution with a log link function for the dependent variable. For rows "DRG FE" and "Hospital FE," an X indicates that the corresponding fixed effects are included in the specification.

A6 Additional Appendix Tables and Figures

Table A5: Final Set of Hospital Inpatient Claims

Steps to Obtain the Final Sample	No. of Claims
Initial BCBS individual plan claims by Arkansas residents	46,074
1. Remove the claims that are not from general acute care hospitals.	
(behavioral hospitals, children hospitals, and specialty hospitals are excluded)	39,719
2. Remove the claims for children and the elderly.	37,033
3. Remove the claims from out-of-network hospitals.	37,012
4. Remove the claims without DRG codes or with invalid DRG codes.	36,929
5. Remove the outlier claims†	36,163
6. Remove hospital-insurer-year triplets with a small number of claims‡	36,036
7. Remove the claims from non-Arkansas hospitals	**34,828**

Notes: This table shows how the final claims set is obtained from the initial claims dataset. "No. of Claims" shows the number of claims remaining after taking each step.
†Outliers are defined as the claims satisfying at least one condition from the following: (1) DRG weight is greater than the 99th percentile, and (2) the allowed amount is greater than the 99th percentile or lower than the 1st percentile.
‡Hospital-insurer-year triplets having more than five claims are only considered. The threshold number of claims does not affect the results of the analysis significantly.

Table A6: Hospital Market Structure in Arkansas

Variable	2013	2014	2015
Number of Hospitals	142	145	143
Number of Hospital Systems	22	22	23
Share of Hospitals Affiliated with Hospital Systems (%)	57.7	57.2	58.0
Hospital System HHI	0.2155	0.2687	0.2499

Sources: (a) AHA Annual Survey, (b) inpatient claims data from the Arkansas APCD, and (c) Hospital System HHI: data is from "Healthy Marketplace Index" by the Health Care Cost Institute (HCCI) (https://healthcostinstitute.org/hcci-originals/hmi-interactive#HMI-Summary-Report-Current-Spending). It represents the Herfindahl-Hirschman Index, constructed using inpatient admissions at the hospital system level for the Little Rock-North Little Rock-Conway Metro Area.

Table A7: Comparing Risk Pools between BCBS ACA and Existing Individual plans

	ACA Individual Plan (2014 and 2015)	Existing Individual Plan (2013)	Difference†
Enrollment (379,968 member-years)			
Mean Enrollee Age	40.6	41.5	-0.9
Share of Enrollee Aged 45 or Older (%)	41.1	44.4	-3.3
Share of Female Enrollees (%)	59.0	48.2	10.8
Inpatient Claims (31,360 claims)			
Mean Patient Age	42.8	44.7	-1.9
Share of Patients Aged 45 or Older (%)	48.8	54.9	-6.1
Share of Female Patients (%)	66.2	56.5	9.7
Mean DRG Weight	1.311	1.370	-0.059
Median DRG Weight	0.985	1.035	-0.050

Source: Author's calculation using the inpatient claims and enrollment data from the Arkansas APCD.
Notes: †The mean (median) difference for each variable is significant at the 1% level according to a bivariate OLS (quantile) regression of the variable against the indicator for ACA individual plan enrollees/patients.

Table A8: List of Major Diagnostic Categories

Rank	MDC	Description	No. of DRGs	Share (%)	Group	Group Share (%)
1	14	Pregnancy, childbirth and the puerperium	15	23.22	D	23.22
2	5	Diseases and disorders of the circulatory system	67	9.82	C	29.34
3	6	Diseases and disorders of the digestive system	61	9.79		
4	8	Diseases and disorders of the musculoskeletal system and connective tissue	85	9.73		
5	4	Diseases and disorders of the respiratory system	40	7.65	B	23.44
6	13	Diseases and disorders of the Female reproductive system	23	5.58		
7	1	Diseases and disorders of the nervous system	68	5.25		
8	7	Diseases and disorders of the hepatobiliary system and pancreas	33	4.96		
9	18	Infectious and parasitic diseases, systemic or unspecified sites	16	4.02	A	24.01
10	11	Diseases and disorders of the kidney and urinary tract	38	3.890		
11	10	Endocrine, nutritional and metabolic diseases and disorders	22	3.886		
12	21	Injuries, poisonings and toxic effects of drugs	20	3.45		
13	9	Diseases and disorders of the skin, Subcutaneous tissue and breast	28	3.12		
14	20	Alcohol/drug use and alcohol/drug induced organic mental disorders	4	1.46		
15	16	Blood, blood forming organs, immunologic disorder	15	1.11		
16	3	Diseases and disorders of the ear, nose, mouth and throat	23	0.77		
17	17	Myeloproliferative diseases and disorders, poorly differentiated neoplasm	25	0.55		
18	12	Diseases and disorders of the male reproductive system	14	0.42		
19	23	Factors influencing health status and other contacts with health services	9	0.39		
20	19	Mental diseases and disorders	8	0.34		
21	24	Multiple significant trauma	7	0.25		
22	25	Human immunodeficiency virus infections	5	0.15		
23	2	Diseases and disorders of the eye	8	0.14		
24	22	Burns	4	0.06		

Source: Descriptions are from the 2014 crosswalk maintained by the National Bureau of Economic Research (NBER). The number of DRGs and shares are based on the author's calculation using the set of BCBS inpatient claims for ACA Medicaid beneficiaries between 2014 and 2015.
Notes: "Group" indicates the group to which each MDC belongs based on the classification described in Table A4. "Group Share (%)" represents the proportion of claims for each MDC group.

References

Brooks, John M, Avi Dor, and Herbert S Wong. 1997. "Hospital-Insurer Bargaining: An Empirical Investigation of Appendectomy Pricing." *Journal of Health Economics*, 16: 417–434.

Capps, Cory, David Dranove, and Mark Satterthwaite. 2003. "Competition and Market Power in Option Demand Markets." *The RAND Journal of Economics*, 34: 737.

Chipty, Tasneem, and Christopher M Snyder. 1999. "The Role of Firm Size in Bilateral Bargaining: A Study of the Cable Television Industry." *The Review of Economics and Statistics*, 81: 326–340.

Crémer, Jacques, and Michael H Riordan. 1987. "On Governing Multilateral Transactions with Bilateral Contracts." *The RAND Journal of Economics*, 18: 436–451.

Gowrisankaran, Gautam, Aviv Nevo, and Robert Town. 2015. "Mergers When Prices are Negotiated: Evidence from the Hospital Industry." *American Economic Review*, 105: 172–203.

Ho, Kate. 2009. "Insurer-Provider Networks in the Medical Care Market." *American Economic Review*, 99: 393–430.

Horn, Henrick, and Asher Wolinsky. 1988. "Bilateral Monopolies and Incentives for Merger." *The RAND Journal of Economics*, 19: 408–419.

Lewis, Matthew S., and Kevin E. Pflum. 2015. "Diagnosing Hospital System Bargaining Power in Managed Care Networks." *American Economic Journal: Economic Policy*, 7: 243–274.

Raskovich, Alexander. 2003. "Pivotal Buyers and Bargaining Position." *The Journal of Industrial Economics*, 51: 405–426.

Snyder, Christopher M. 1996. "A Dynamic Theory of Countervailing Power." *The RAND Journal of Economics*, 27: 747–769.

Stole, Lars A, and Jeffrey Zwiebel. 1996. "Intra-Firm Bargaining under Non-Binding Contracts." *The Review of Economic Studies*, 63: 375–410.
